# UBE2O ubiquitinates PTRF/CAVIN1 and inhibits the secretion of exosome-related PTRF/CAVIN1

**DOI:** 10.1186/s12964-022-00996-z

**Published:** 2022-11-28

**Authors:** Xiaotong Cen, Qing Chen, Bin Wang, Hongjie Xu, Xu Wang, Yixia Ling, Xiaofei Zhang, Dajiang Qin

**Affiliations:** 1 Key Laboratory of Biological Targeting Diagnosis, Therapy and Rehabilitation of Guangdong Higher Education Institutes, The Fifth Affiliated Hospital of Guangzhou Medical University, Guangzhou, Guangdong 510799 China; 2 Basic Research Center, BioLand Laboratory (Guangzhou Regenerative Medicine and Health Guangdong Laboratory), Guangzhou, Guangdong 510530 China; 3 Centre for Regenerative Medicine and Health, Hong Kong Institute of Science & Innovation, Chinese Academy of Sciences, Hong Kong SAR, China; 4 CAS Key Laboratory of Regenerative Biology, Guangdong Provincial Key Laboratory of Stem Cell and Regenerative Medicine, Center for Cell Lineage and Development, GIBH-HKU Guangdong-Hong Kong Stem Cell and Regenerative Medicine Research Centre, Guangzhou Institutes of Biomedicine and Health, Chinese Academy of Sciences, Guangzhou, Guangdong 510530 China

**Keywords:** Exosome secretion, PTRF/CAVIN1, SDPR/CAVIN2, UBE2O, Ubiquitination

## Abstract

**Background:**

Exosomes are small vesicles released by cells, which have crucial functions in intercellular communication. Exosomes originated from cell membrane invagination and are released followed by multivesicular bodies (MVBs) fused with the cell membrane. It is known that Polymerase I and Transcript Release Factor (PTRF, also known as Caveolin-associated Protein-1, CAVIN1) plays an important role in caveolae formation and exosome secretion. And PTRF in exosomes has been identified as a potential biomarker in multiple malignancies such as glioma and renal cell carcinoma. However, the mechanisms of how to regulate the secretion of exosome-related PTRF remain unknown.

**Methods:**

We performed exogenous and endogenous immunoprecipitation assays to investigate the interaction between ubiquitin-conjugating enzyme E2O (UBE2O) and PTRF. We identified UBE2O ubiquitinated PTRF using ubiquitination assays. Then, exosomes were isolated by ultracentrifugation and identified by transmission electronic microscopy, western blot and nanoparticle tracking analysis. The effect of UBE2O on the secretion of exosome-related PTRF was analyzed by western blot, and the effect of UBE2O on exosome secretion was evaluated by exosome markers and the total protein content of exosomes.

**Results:**

Here, we showed that UBE2O interacts with PTRF directly and ubiquitinates PTRF. Functionally, we found that UBE2O inhibited the effects of PTRF on exosome secretion via decreasing caveolae formation. Importantly, UBE2O decreased exosome secretion, resulting in downregulating PTRF secretion via exosomes. Our study also identified Serum Deprivation Protein Response (SDPR, also known as Caveolin-associated Protein-2, CAVIN2) interacted with both UBE2O and PTRF. Furthermore, we found that SDPR promotes PTRF expression in exosomes. Interestingly, even in the presence of SDPR, UBE2O still inhibited the secretion of exosome-related PTRF.

**Conclusions:**

Our study demonstrated that UBE2O downregulated exosome release and controlled the secretion of exosome-related PTRF through ubiquitinating PTRF. Since exosomes play an important role in malignant tumor growth and PTRF included in exosomes is a biomarker for several malignant tumors, increasing UBE2O expression in cells has the potential to be developed as a novel approach for cancer treatment.

**Video Abstract**

**Supplementary Information:**

The online version contains supplementary material available at 10.1186/s12964-022-00996-z.

## Background

Exosomes, small extracellular vesicles with the size in 30–150 nm, are released by most cell types to the extracellular environment [1]. The invagination of cell membranes is the beginning step for the biogenesis of exosomes, followed by the formation of early endosomes. Multivesicular bodies (MVBs), the late endosomes with the presence of intraluminal vesicles (ILVs), are transported to and fuse with the plasma membrane to release the ILVs as exosomes [2, 3]. Exosomes selectively carry biological cargo, such as protein, coding RNA, non-coding RNA, DNA, proteins, lipids and metabolites [4–6], to mediate intercellular communications locally and distally. Exosomes have critical roles in modulating the tumor microenvironment, including proangiogenic effect [7, 8] and immunoregulation [9–11], hence the proliferation and metastasis of malignant tumors are closely associated with exosomes [12, 13]. Recently, Lin et al. showed that HeLa cell-derived exosomes induce endoplasmic reticulum stress in endothelial cells, resulting in vascular integrity breakdown and tumor metastasis [14]. Based on the functions of exosomes in tumors, exosomes can be a target for cancer treatment [15]. Reduced secretion of exosomes inhibits tumor development [16]. Moreover, exosome-base biomarkers for early diagnosis have been focused on [17–19]. However, to study how to regulate proteins in exosomes, including exosome biomarkers of tumors, is still needed.

Caveolae are small invagination (50–100 nm) formed from the plasma membrane that plays critical roles in cellular processes such as endocytosis and signal transduction. Because exosomes are derived from the invagination of cell membranes, caveolae are related to exosome biogenesis [18]. Both Polymerase I and Transcript Release Factor (PTRF, also known as Caveolin-associated Protein-1, CAVIN1) and Serum Deprivation Protein Response (SDPR, also known as Caveolin-associated Protein-2, CAVIN2) are the members of CAVIN family, which have important functions in caveolar biogenesis [20]. It is known that PTRF is involved in caveolae formation [21–25], while SDPR is essential for caveolar membrane curvature [26]. Recently, Huang et.al demonstrated that PTRF induces exosome secretion in human glioma cells and serves as a biomarker of both glioma and serum exosomes [18]. In addition, SDPR regulates the stability of PTRF in HeLa cells and recruits PTRF to caveolae via directly interacting with PTRF [26, 27].

Ubiquitin-conjugating enzyme E2O (UBE2O) contains 1292 amino acids, is a large E2 ubiquitin-conjugation enzyme. Previous studies have found that UBE2O functions as a hybrid E2/E3 ubiquitin-protein ligase [28, 29]. UBE2O regulates signaling pathways such as NF-κB and BMP via regulating the ubiquitination of key regulators of these pathways [30, 31]. It is found that the ubiquitination and degradation of protein modulated exosome release [32]. Interestingly, UBE2O mediates the ubiquitination and degradation of various proteins in different types of cancer. These proteins include transcription factor c-Maf [33], AMP-activated protein kinase-α2 (AMPKα2) [34–36], mixed-lineage leukemia (MLL) [37], tumor suppressor BAP1 [38] and aryl hydrocarbon receptor nuclear translocator-like protein 1 (ARNTL or BMAL1) [39]. However, the role of UBE2O in exosome secretion is still unknown.

Previously, we identified PTRF and SDPR as UBE2O interactors from a mass-spectrometry based interactome study of UBE2O [40]. In this study, we used immunoprecipitation to validate the interaction of UBE2O with both PTRF and SDPR. We showed that UBE2O ubiquitinates and promotes the degradation of PTRF, leading to the decrease of exosome-related PTRF secretion. The inhibition of exosome-related PTRF secretion by UBE2O is not eliminated by the overexpression of SDPR. In addition, we found that UBE2O-mediated PTRF degradation decreases caveolae formation and exosome secretion. In summary, our results provide molecule evidences of how UBE2O participates in exosome biogenesis and how UBE2O regulates the secretion of exosome-related PTRF.

## Materials and methods

### Plasmids

Both full-length human PTRF and SDPR from cDNA of HeLa cells were cloned into pCR3.1-Myc and lentivirus vector pLV-Flag. Plasmids including pCR3.1-UBE2O-Myc, pCR3.1-UBE2O-C1040S-Myc, His-HA-Ubi, His-HA-Ubi-KO and pLV-Flag-UBE2O had been described in the previous studies [30, 40]. UBE2O deletion constructs (D1–D5), PTRF deletion constructs (D1–D3) and SDPR deletion constructs (D1–D2) were cloned into pLV-Flag vector. PTRF deletion constructs D3 was also cloned into pGEX-5X-1 vector. And the truncated version of the conserved region 2 (CR2) of UBE2O was cloned into pET28A vector. All the primers for UBE2O deletion constructs D1–D5, PTRF deletion constructs D1–D3, SDPR deletion constructs D1–D2 and the truncated version of UBE2O CR2 were shown in Additional file 1: Table S1. With regard to the construction of vectors for PTRF and SDPR knockdown, PTRF or SDPR shRNA forward primer and reverse primer were complementary paired and followed by inserted into an inducible shRNA lentivirus vector. The primers for four PTRF shRNA sequences and three SDPR shRNA sequences also can be found in Additional file 1: Table S1. All plasmids were verified by DNA sequencing.

### Cell culture, transfection and stable cell lines construction

HEK293T and HeLa cells, obtained from ATCC, were cultured in DMEM/High glucose (HyClone, SH30022.01) supplemented with 10% fetal bovine serum (FBS, Gibco, 10270-106) and 100 U/ml penicillin/streptomycin (HyClone, SV30010). Cells were cultured in the condition of 37 °C and 5% CO_2_, and had been tested for mycoplasma (Lonza, LT07-318).

Plasmids were transfected into cells by transfection reagent poly-ethylenimine 25 K (PEI 25 K, Polysciences, Inc, 23966-100). After 36 h transfection, cells were collected for analysis by immunoprecipitation assays or ubiquitination assays.

Lentiviruses were produced by transfecting virus packaging plasmids pRSV-REV, pCMV-VSVG and pMDLg-RRE (gag/pol) with the desired plasmid into HEK293T. Cell supernatants collected after 48 h transfection were used to infect HeLa cells with 8 μg/ml of polybrene (Sigma). Cells were selected by 200 μg/ml hygromycin B (Sigma, V900372-250MG) or 1 μg/ml puromycin (Sigma, P8833-10MG) after at least 2 days infection. For UBE2O knockout cell lines, UBE2O sgRNA forward primer caccgcatctatcccgtcaacagca and UBE2O sgRNA reverse primer aaactgctgttgacgggatagatgc were cloned into pSpCas9(BB)-2A-GFP [41]. Cells transfected with sgRNA expression plasmid were sorted by fluorescence-activated cell sorter (MoFlo Astrios, USA) 48 h after transfection. Single cells were re-seeded and cultured with the normal medium in the presence of 1/1000 TG/BCS (0.433% α-Thioglycerol (α-TG, Sigma, M6145), 20 μM bathocuproinedisulfonic acid disodium salt (BCS, Sigma, B1125)). UBE2O knockout cell lines were verified by western blot and DNA sequencing.

### Immunoprecipitation assays

For immunoprecipitation assays, cells were lysed with WCE lysis buffer (10% glycerol, 150 mM NaCl, 50 mM Tris–HCl, pH 8.0, 0.5%NP40) plus 1 × protease inhibitor cocktail (Bimake, B14001) on ice for 20 min. Cell lysates were centrifuged at 14,000 rpm, 4 °C for 10 min. 50 μl supernatant was aliquoted as input, and Flag M2 beads were used for immunoprecipitation (Sigma, M8823) at 4 °C for 1.5 h. For endogenous immunoprecipitation assays, cell lysates were incubated with PTRF antibody (Proteintech, 18892-1-AP) or UBE2O (Bethyl Laboratories, A301-873A) overnight. Then, the cell lysates with PTRF antibody were incubated with protein A resin (GenScript, L00210) for 2 h, and the cell lysates with UBE2O antibody were incubated with protein A/G magnetic beads (MedChemExpress, HY-K0202) for 2 h. After the incubation, beads which included Flag, protein A or protein A/G beads were washed by WCE lysis buffer for three times. Both the input samples and the immunoprecipitated samples were boiled with 2 × SDS loading buffer at 95 °C for 10 min. All the boiled samples were analyzed by western blot.

### Ubiquitination assays

Cells were washed twice with cold phosphate-buffered saline (PBS, BI, 02-024-1ACS) with 10 mM *N*-Ethylmaleimide (NEM, Sigma, E3876-5G) and lysed with 8 M urea buffer solution (8 M urea, 10 mM imidazole, 10 mM Tris–HCl pH 8.0, 0.1 M NaH_2_PO_4_, 0.1 M Na_2_HPO_4_) with 10 mM β-mercaptoethanol (Macklin, M828395-100 ml). Lysates were centrifuged at 14,000 rpm, room temperature for 10 min. The supernatant was incubated with nickel resins (GenScript, L00223I-50) at room temperature for 2 h. Nickel beads were washed 3 times by 8 M urea buffer solution with 20 mM imidazole, and protein was eluted with 2 × SDS loading buffer.

### Purification of PTRF-D3 and UBE2O-CR2

Protein expression was performed using BL21 strain of *E. coli* (Vazyme, C504-02) with plasmids of pGEX-5X-1, pGEX-5X-1-PTRF-D3 and pET28A-UBE2O-CR2. GST protein was induced at 37 °C with 0.25 mM isopropyl thiogalactoside (IPTG) for 6 h, GST-PTRF-D3 was induced at 25 °C with 0.25 mM IPTG overnight, and His-UBE2O-CR2 was induced at 37 °C with 0.25 Mm IPTG for 6 h. Bacteria were collected at 4000 rpm, 4 °C for 15 min and were re-suspend by PBS, lysozyme (Macklin, L6051-25 g) and benzonase nuclease (Sigma, E1014-25KU). Follow by ultrasound pyrolysis, samples were centrifuged at 14,000 rpm, 4 °C for 10 min. The supernatant was filtered by the 0.45 μm filter (Millipore, SLHV033RB). Lysates with GST or GST-PTRF-D3 protein were incubated with glutathione resin (GenScript, L00206-100) and eluted by 10 mM L-glutathione (Biofroxx, YS-1392GR005), while lysates of His-UBE2O-CR2 protein were incubated with nickel resins and eluted by 50 mM imidazole eluent (50 mM Tris–HCl, pH 8.0, 0.15 M NaCl, 50 mM imidazole). All eluted proteins were finally concentrated by the 10 K centrifugal filter (Millipore, UFC801008).

### In vitro interaction

His-UBE2O CR2 protein was incubated with nickel resins at 4 °C for 1.5 h. Followed by three washed with WCE lysis buffer, GST or GST-PTRF-D3 protein was added to immunoprecipitates and was incubated at 4 °C overnight. Nickel beads were washed with WCE lysis buffer for three times and boiled with 2 × SDS loading buffer for western blot.

### Exosomes isolation

Approximately 5 × 10^6^ cells were seeded in cell culture dishes. To make the data more accurate, the same number of cells were seeded in different comparison groups each time. Then, cells were cultured in normal medium until 80% confluent, followed by 3 PBS washes and 18 mL of serum-free medium culturing for an additional 40 h. Next, the serum-free conditioned medium was harvested and centrifuged sequentially at 300 × g and 2000 × g for 10 min to remove cells and cell debris, respectively. Small cell debris and larger microvesicles were removed by 10,000 × g centrifugation for 30 min (Avanti J-E, Beckman, USA). Finally, the exosome-rich pellet was collected after ultracentrifugation at 167,000 × g for 70 min (XPN-100, Beckman, USA). The exosome-rich pellet was washed by PBS and ultracentrifuged at 167,000 × g for 70 min again [42, 43]. Exosomes were aliquoted for the following experiments.

### Western blot and BCA assay

For the quantification of total protein content in exosomes, exosomes and their host cells were collected and were lysed by RIPA lysis buffer (Beyotime, P0013K). To ensure exosomes were secreted by the similar number of cells in the different comparison group, the detection amount of exosome lysates was adjusted according to the total protein content of their host cells. The total protein content of exosomes was quantified by the Pierce BCA protein assay kit (Thermo, 23227).

For western blot, protein concentrations of cell lysates were also quantified by the Pierce BCA protein assay kit according to product instructions. Equal amounts of protein samples were boiled in 1 × SDS loading buffer and resolved by SDS-PAGE. Proteins were transferred to the PVDF membrane (Millipore, ISEQ00010) at 50 mA overnight (Bio-Rad, USA). Membrane was incubated in blocking buffer (5% skim milk powder (Biofroxx, 1172GR500) in 1 × TBST, 50 mM Tris–Cl, pH 7.6, 150 mM NaCl, 0.1% Tween 20) for 1 h. Then, the membrane was incubated in the primary antibodies for 2 h and in the secondary antibody for 1 h at room temperature. Western blot was visualized using ChemiDoc Imaging systems (Bio-Rad, USA). Blots were quantified by ImageJ.

Antibodies used in this study included PTRF (Proteintech, 18892-1-AP), SDPR (Proteintech, 12339-1-AP), Flag M2 (Sigma-Aldrich, F1804), Flag (Sigma-Aldrich, F7425-2MG), GST (Invitrogen, PA1-982A), UBE2O (Invitrogen, PA5-54839), UBE2O (Bethyl Laboratories, A301-873A), HA (Sigma-Aldrich, H3663-200UL), Myc (Proteintech, 16286-1-AP), His (Santa Cruz, sc-8036), TSG101 (Abcam, ab125011), CD63 (Proteintech, 25682-1-AP), CD9 (Proteintech, 20597-1-AP), Apolipoprotein A1 (Abcam, ab52945), GM130 (Abcam, 52649), Calnexin (Proteintech, 10427-2-AP), GAPDH (KANGCHE, KC-5G5), Goat Anti-Mouse IgG HRP (KANGCHE, KC-MM-035), Goat Anti-Rabbit IgG HRP (KANGCHE, KC-RB-035), and IPKine™ HRP, Mouse Anti-Rabbit IgG LCS (Abbkine, A25022).

### Nanoparticle tracking analysis (NTA)

Exosome samples were diluted in PBS for analysis. Exosome size distribution was measured by ZetaView PMX 110 (Particle Metrix, Meerbusch, Germany) and analyzed by ZetaView 8.04.02 SP2 software.

### Transmission electronic microscopy (TEM)

For TEM analysis, cells were fixed with 3% glutaraldehyde for at least 4 h. After being washed by PBS for three times, samples were post-fixed using 1% osmic acid for 2 h. Then, samples were washed by PBS for three times again and sequentially dehydrated in 50%, 70%, 80% and 90% alcohol for 15 min. Next, samples were dehydrated in 100% alcohol and 100% acetone twice for 15 min, respectively. After samples were embedded using epoxy resin to form small blocks, ultrathin sections (100 nm) of samples were harvested by an ultramicrotome (UC7, Leica, Germany). Ultrathin sections were stained with uranyl acetate for 20 min and were stained with lead citrate for 15 min. Finally, samples were imaged by TEM (Tecnai G2 Spirit, USA).

For TEM analysis of exosomes, the isolated exosomes (10 μl) were fixed in 4% paraformaldehyde (PFA, Beyotime, P0099-500 ml), then were dropped onto formvar-carbon-coated grids. After 5 min, the excess fluid in grids was removed. Next, samples were stained with 3% phosphotungstic acid for 5 min. Finally, samples were air-dried and were analyzed by TEM (Tecnai G2 Spirit, USA).

### Confocal imaging

Cells on coverslips were fixed by 4% PFA at room temperature for 20 min and washed by PBS with 2 mg/ml glycine (GenStar, VA13110-500 g). Then, samples were treated with 0.3% Triton X-100 for 10 min and blocked by 10% FBS for 1 h at room temperature. After being washed by PBS, samples were incubated in primary antibody which was diluted in PBS containing 2% FBS for 1.5 h at room temperature. Next, samples were incubated in secondary antibody followed by 4’, 6-diamidine-2-phenylindole (DAPI, Cell Signaling, 8961S). Finally, coverslips were mounted onto slips using fluorescence mounting medium (Dako, S3023) and fluorescent images were taken by immunofluorescence microscope (Zeiss 710 NLO, Germany).

Antibodies used for immunofluorescence included Flag M2 (Sigma-Aldrich, F1804), PTRF (Proteintech, 18892-1-AP), Alexa Fluor 568 goat anti-mouse IgG (Invitrogen, A11004), Alexa Fluor 488 goat anti-rabbit IgG (Invitrogen, A11008).

### RNA extraction and real-time PCR (RT-PCR) analysis

Total RNA was isolated from HeLa cells using TRIzol reagent (MRC, TR118-200). Complementary DNA (cDNA) was synthesized from the reverse transcription of 900 ng of total RNA using HiScript® II Q RT SuperMix for qPCR (Vazyme, R222-01) according to the product instruction. ChamQ SYBR qPCR Master Mix (Vazyme, Q311-02/03) was used for RT-PCR. Samples were detected by a CFX96 Touch™ RT-PCR Detection System (Bio-Rad, USA). All primers used for RT-PCR were listed in Additional file 1: Table S1.

### Statistical analysis

Statistical analyses were performed as the means ± SEM by GraphPad Prism 5. *P* values for differences were calculated using Student’s *t*-test. *P* $<$ 0.05 was considered to a statistically significant difference.

## Results

### UBE2O directly interacts with PTRF

**Fig. 1:**
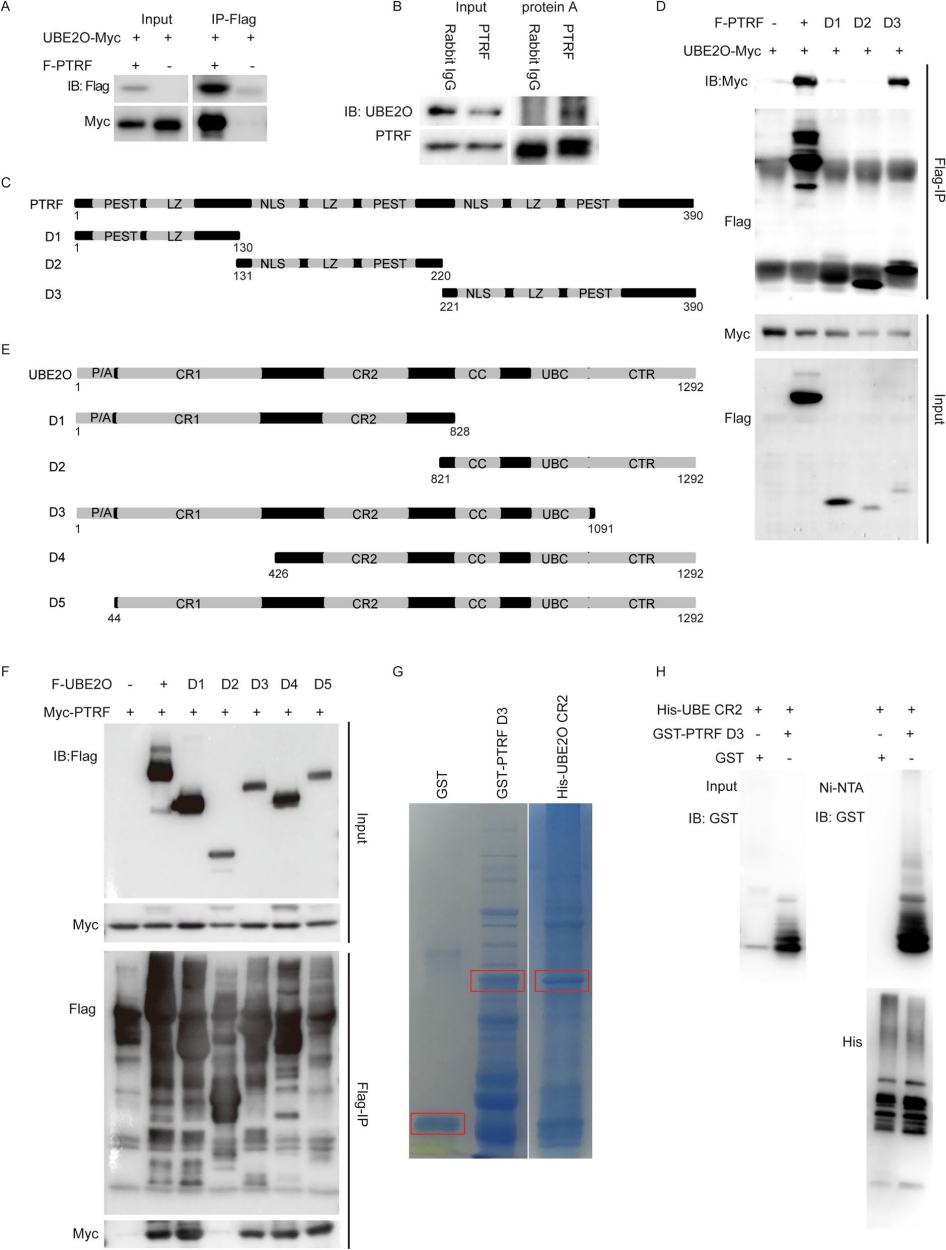
UBE2O combines with PTRF directly. **A** PTRF co-immunoprecipitates UBE2O. Myc-UBE2O and Flag-PTRF expression plasmids were co-transfected into HEK293T cells by using PEI transfection. Cell lysates were prepared after 40 h transfection, and Flag-PTRF in the cell lysates combined with FLAG M2 beads. The immunoprecipitates and the total cell lysates were analyzed by immunoblotting (IB) as shown. **B** Endogenous PTRF in HeLa cells was immunoprecipitated with PTRF antibody by protein A. PTRF-associated endogenous UBE2O was detected by IB. **C** Wild-type PTRF and PTRF deletion mutants D1, D2 and D3 were shown as schematic representations. **D** UBE2O-Myc and Flag-PTRF or Flag-PTRF deletion constructs (D1–D3) vectors were transfected into HEK293T cells. Flag M2 beads immunoprecipitates and the total cell lysates were immunoblotted with Myc and Flag antibodies. **E** Wild-type UBE2O and UBE2O deletion mutants D1, D2, D3, D4 and D5 were shown as schematic representations. **F** Myc-PTRF and Flag-UBE2O or five UBE2O deletion vectors (D1–D5) were transfected into HEK293T cells. Cell lysates were analyzed by IP and IB as indicated. **G** GST and GST-PTRF D3 were purified on GST-agarose, and His-UBE2O CR2 was purified on Ni-agarose. The framed strips were the purified protein strips. **H** The purified GST or GST-PTRF D3 was applied to the purified His-UBE2O CR2 immobilized on Ni–NTA superflow as indicated. Eluted proteins were analyzed by western blotting

We previously showed that PTRF is a UBE2O interactor using mass-spectrometry based interactome study [40]. To validate the interaction between UBE2O and PTRF, UBE2O-Myc vectors were transfected into HEK293T cells with or without Flag-PTRF vectors. As shown in Fig. 1A, UBE2O-Myc was immunoprecipitated in transfection condition with Flag-PTRF but not in the condition of control. To further confirm their interaction in the endogenous level, cell lysates were immunoprecipitated with PTRF antibody (Fig. 1B). In line with the ectopic results, endogenous UBE2O was immunoprecipitated by PTRF antibody. According to the method of Gould et al. to choose a PTRF high cell line and a PTRF low cell line [44], the expression level of PTRF in several cell lines was detected by western blot. The results of western blot showed that the expression level of PTRF in U-2 OS cells was lower than that in HeLa cells, while the expression level of PTRF in U251 cells was higher than that in HeLa cells (Additional file 2: Figure S1A). The endogenous immunoprecipitation assay was also performed in U-2 OS cells and U251 cells, showing that UBE2O interacted with PTRF endogenously, which was the same as the results in HeLa cells (Additional file 2: Figure S1B and C). To map the domain of PTRF that is responsible for the interaction with UBE2O, three deletion constructs (D1–D3) of PTRF were constructed according to their functional domain (Fig. 1C). Co-immunoprecipitation assays displayed that UBE2O-Myc was mainly immunoprecipitated by PTRF deletion mutant D3, which contains nuclear localization sequence (NLS), leucine-zipper motif (LZ) and PEST sequence (PEST) (Fig. 1D). Next, five UBE2O deletion mutates (D1–D5) with Flag tag were used to study their interaction with PTRF (Fig. 1E). As shown in Fig. 1F, all UBE2O mutations except D2 which lacks the CR2 domain interacted with PTRF, indicating that the CR2 domain is responsible for the interaction of UBE2O and PTRF. Moreover, we showed the direct interaction between the CR2 domain of UBE2O and D3 of PTRF using recombinantly expressed proteins (Fig. 1G, H). Therefore, UBE2O combines with PTRF directly via the CR2 domain.

### UBE2O ubiquitinates PTRF via both monoubiquitination and polyubiquitination

**Fig. 2:**
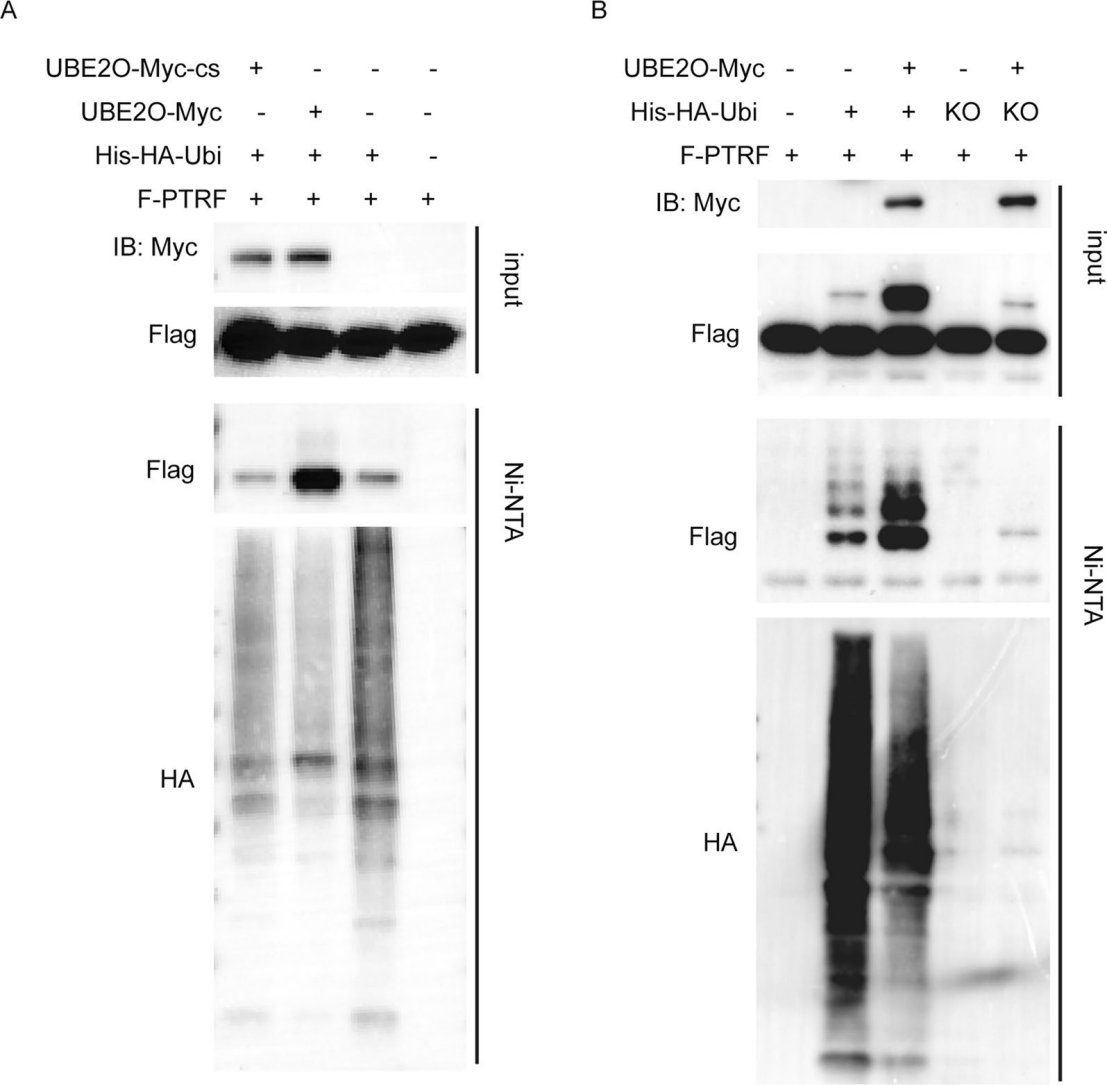
UBE2O ubiquitinates PTRF. **A** UBE2O-Myc-cs expression plasmid is a UBE2O mutation expression plasmid. UBE2O expressed by the UBE2O-Myc-cs expression plasmid could not bind to ubiquitin effectively. Flag-PTRF, His-HA-ubiquitin and UBE2O-Myc or UBE2O-Myc-cs expression plasmids were co-transfected into HEK293T cells. After cells were collected, His-HA-ubiquitin in cell lysates was immunoprecipitated with Ni–NTA superflow. The immunoprecipitates and the total cell lysates were immunoblotted for the indicated antibodies. **B** Flag-PTRF, UBE2O-Myc and His-HA-ubiquitin-KO or His-HA-ubiquitin wild type vectors were co-transfected into HEK293T cells. Cells were harvested after 40 h transfection and were analyzed by His immunoprecipitation ubiquitination assays and western blot

Since UBE2O interacts with PTRF directly and UBE2O functions as a hybrid E2/E3 ubiquitin-protein ligase, PTRF ubiquitination assays were performed. His-Ubiquitin-associated PTRF was strongly immunoblotted after nickel pull-down assays in the condition with co-transfection of His-HA-ubiquitin and UBE2O-Myc (Fig. 2A). Thus, UBE2O ubiquitinates PTRF. Then, His-HA-Ubi-KO plasmids were transfected into HEK293T cells with UBE2O-Myc and Flag-PTRF plasmids. His-HA-Ubi-KO was ubiquitin mutant which could not form polyubiquitin linkage. After ubiquitination assays, we found that multiple strong UBE2O-mediated ubiquitinylation bands were seen in the group which had been transfected His-HA-Ubi, while there was only one band in the group transfected with His-HA-Ubi-KO (Fig. 2B). Thus, UBE2O could both monoubiquitinated and polyubiquitinated PTRF.

### UBE2O interacts with SDPR via its CR2 domain

**Fig. 3:**
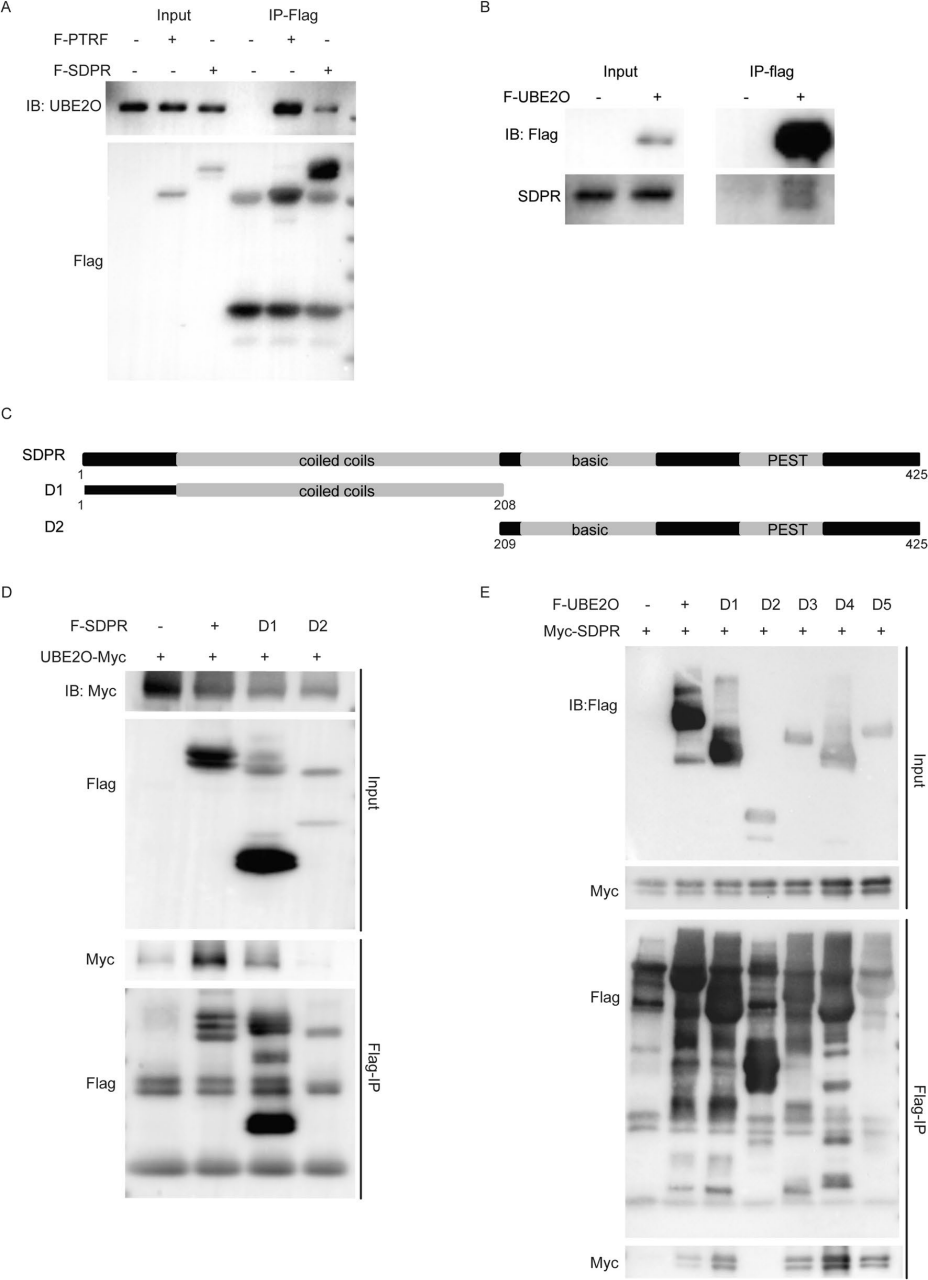
The CR2 domain in UBE2O interacts with SDPR. **A** Flag-PTRF or Flag-SDPR expression plasmid was infected into HeLa cells. After cell lysates were prepared, Flag-PTRF or Flag-SDPR in cell lysates was immunoprecipitated with Flag M2 beads. PTRF-associated endogenous UBE2O or SDPR-associated endogenous UBE2O was detected by IB. **B** HeLa cells were transiently transfected with Flag-UBE2O vector. Flag-UBE2O-associated endogenous SDPR was detected by IP and IB. **C** Wild-type SDPR and SDPR deletion mutants D1 and D2 were shown as schematic representations. **D** UBE2O-Myc and Flag-SDPR or Flag-SDPR deletion constructs (D1–D2) vectors were transfected into HEK293T cells. After 40 h transfection, cells were harvested for IP and IB analyses. **E** Flag-SDPR and five UBE2O deletion constructs (D1–D5) were expressed in HEK293T cells. After cell lysates were precipitated with Flag M2 beads, immunoprecipitates and the total cell lysates were immunoblotted with the indicated antibody as shown

Our previous UBE2O interactome results also identified SDPR [40]. Because the commercially available antibodies for UBE2O and SDPR are not suitable for immunoprecipitation assay, we alternatively validated these interactions via using semi-endogenous immunoprecipitation assays. As shown in Fig. 3A, endogenous SDPR showed obvious interaction with UBE2O as same as PTRF. In addition, the reciprocal interaction assay showed consistent result (Fig. 3B). To map the domain responsible for the interaction of UBE2O and SDPR, we constructed two deletions of SDPR. As shown in Fig. 3C, D1 of SDPR which contains the coiled coils domain interacts with UBE2O-Myc (Fig. 3D). Similar to the interaction with PTRF, UBE2O utilizes the CR2 domain to interact with SDPR (Fig. 3E).

### UBE2O downregulates the secretion of exosome-related PTRF and decreases exosome release

**Fig. 4:**
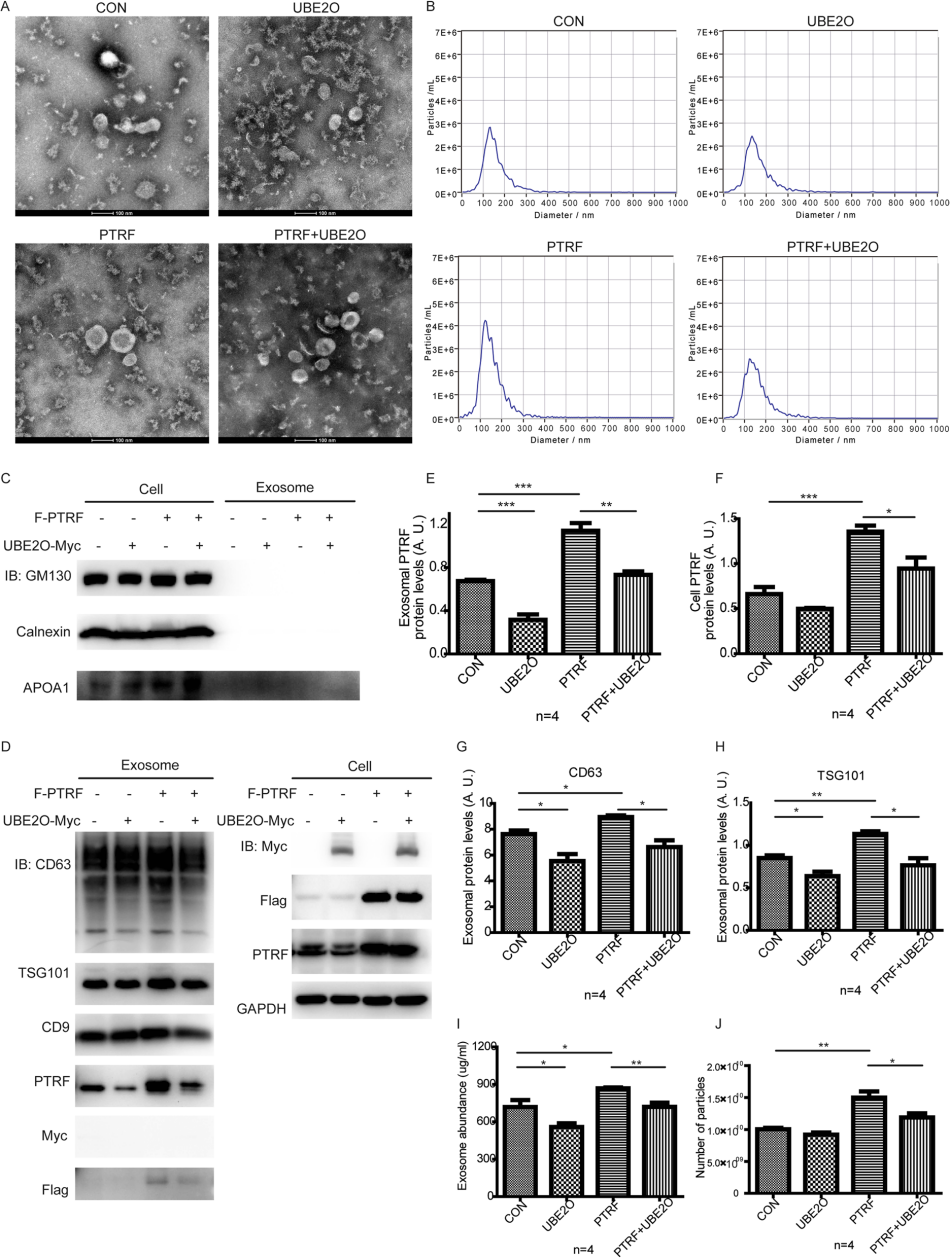
UBE2O controls the secretion of exosome-related PTRF and inhibits exosome secretion. **A** UBE2O was transiently transfected into PTRF or SDPR stable HeLa cell line with PEI. Transmission electron microscopy images of exosomes derived from overexpression HeLa cells were shown as indicated. Scale bar: 100 nm. **B** Exosomes from four groups of overexpressed cells were diluted in the same fold (1:1000). Then, the size distribution of exosomes was identified with nanoparticle tracking analysis (NTA). **C**, **D** HeLa cells were stably transfected with Flag-PTRF and transiently transfected with UBE2O-Myc. After 40 h of cell culture, exosomes in the cell supernatants were extracted. Exosome components and overexpressed cell components were analyzed by western blot. **E**, **F**, **G**, **H** Histograms showed densitometry analyses of exosomal PTRF (**E**), cell PTRF (**F**), CD63 (**G**) and TSG101 (**H**) expression relative to cell GAPDH expression. **I** Total protein of exosomes from four groups of overexpressed cells was measured by BCA assay. **J** The number of particles which with the size in 30–150 nm was determined by NTA. All statistical data are the means $\pm$ SEM from more than 3 independent experiments. *P $<$ 0.05; **P $<$ 0.01; ***P $<$ 0.001

Our results showed that UBE2O ubiquitinates PTRF. In addition, UBE2O was shown to regulate the stability of PTRF during terminal erythroid differentiation [45]. To address whether UBE2O regulates the stability of PTRF in exosomes, we purified exosomes from cells with indicated overexpressed proteins (Fig. 4A). We analyzed the quality of isolated exosomes from different conditions using transmission electron microscopy (TEM) (Fig. 4A) and nanoparticle tracking analysis (NTA) (Fig. 4B). TEM analysis showed that the isolated exosomes from different conditions have normal spherical structure as well as a lipid bilayer, and NTA analysis showed that most of isolated extracellular vesicles are in the line with the size of exosomes. Then, exosomes were lysed by RIPA lysis buffer and were analyzed by western blot. According to the guidelines of MISEV 2018, three negative exosome markers and three positive exosome markers were used to verify the exosome samples [46]. As shown in Fig. 4C and D, apolipoprotein A1 (APOA1), endoplasmic reticulum marker calnexin and Golgi marker GM130 were not included in samples, while all samples from different groups expressed exosome markers CD9, CD63 and TSG101. Moreover, exosome-related PTRF was obviously downregulated by UBE2O, whereas the expression of PTRF was only slightly decreased in HeLa cells with UBE2O was overexpressed (Fig. 4D, E, F). A recent study from Huang, et al. showed that PTRF promotes exosome release [18]. To further explore the influences of UBE2O in the total exosomes, we first analyzed the expression of exosome markers in the condition of PTRF overexpression and obtained consistent results in CD63 and TSG101. Then, we found that UBE2O overexpression decreases the expression of CD63 and TSG101 in exosomes screted from both empty cells and PTRF stable cell line (Fig. 4D, G, H). However, there were no significant changes in the content of CD9 in exosomes with the overexpression of UBE2O or PTRF, which illustrated that UBE2O might mediate PTRF to modulate exosome secretion mainly via CD63 and TSG101 (Fig. 4D, Additional file 2: Figure S2A). In addition, UBE2O was not in the way of changing RNA level in cells to regulate the content of CD63 and TSG101 in exosomes (Additional file 2: Figure S2B, C, D, E, F). Next, we also analyzed the total protein abundances of exosomes from different conditions. As shown in Fig. 4I, PTRF overexpression caused an increase in the total protein content of exosomes, compared to the control, whereas UBE2O overexpression inhibits the total protein content of exosomes. Importantly, we showed that UBE2O also inhibits the upregulation effects of PTRF on the total protein content of exosomes (Fig. 4I). Since the size of exosomes is mainly in 30–150 nm, we then measured the concentration of exosomes using NTA and calculated the number of exosomes which with the size in 30–150 nm. We found that PTRF overexpression also increased the number of exosomes, while UBE2O rescued the effects of PTRF on the number of exosomes. Overall, these results indicated that UBE2O had functions to regulate the secretion of exosome-related PTRF. Since UBE2O decreased the effects of PTRF on the expression of exosome markers, the total protein abundances of exosomes and the number of exosomes, UBE2O inhibited exosome release via PTRF. In addition, these results indirectly demonstrated that UBE2O decreased PTRF-loaded exosome secretion.

### UBE2O inhibits the effects of PTRF on caveolae formation

**Fig. 5:**
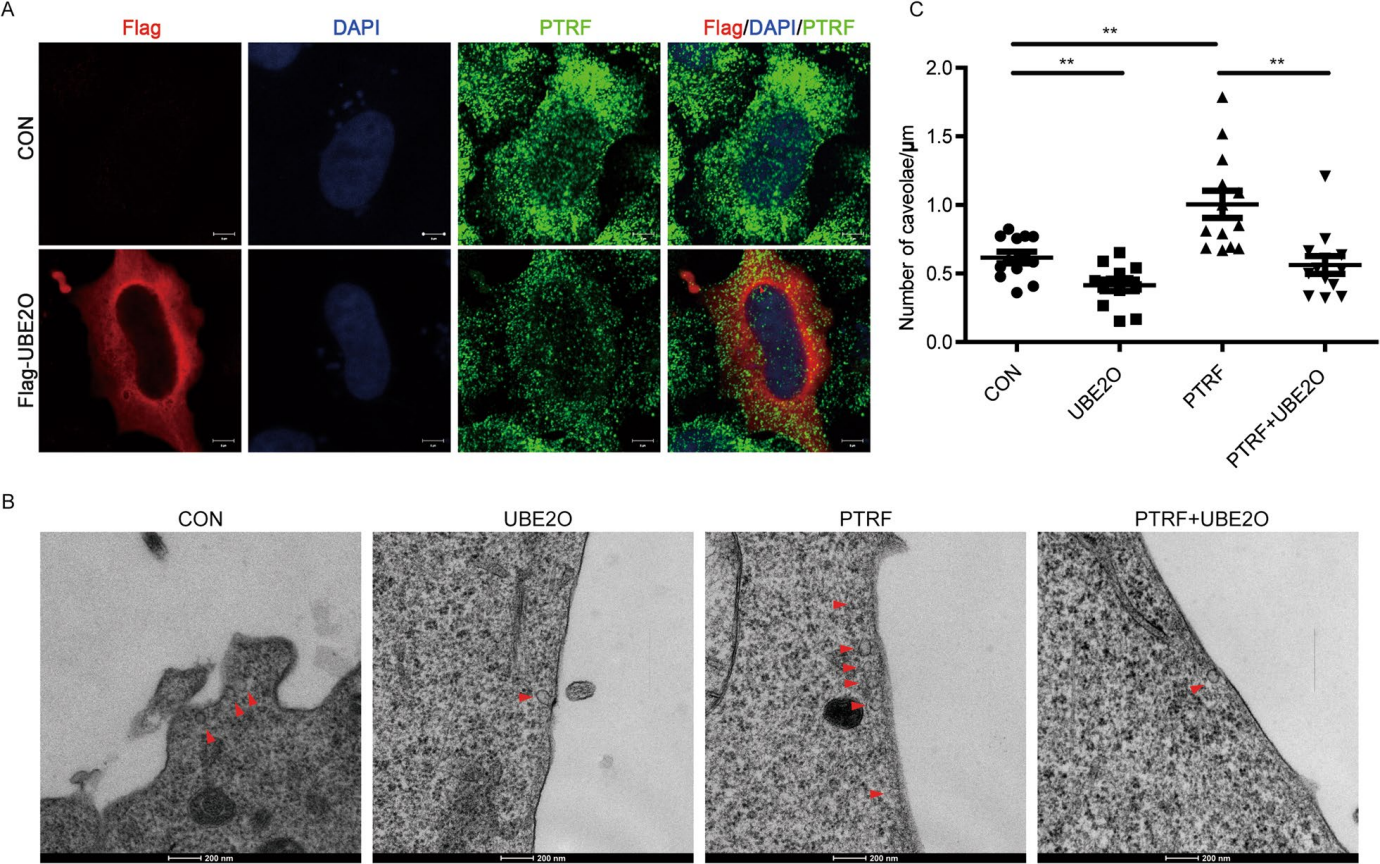
UBE2O limits caveolae formation. **A** Flag-UBE2O was transfected into HeLa cells. After 40 h transfection, HeLa cells were immunostained with an anti-Flag antibody and anti-PTRF antibody. **B**, **C** UBE2O-Myc was transfected into Flag-PTRF stable HeLa cell line. Caveolae structures in HeLa cells were identified by transmission electron microscopy (TEM) scanning. **B** Red arrowhead: caveolae, Scale bar: 200 nm. The number of caveolae per micrometer of the plasma membrane was quantitated through 13 cells analysis. **C** *P* values were calculated with Student’s *t*-test. ***P $<$ 0.001

Next, we used immunofluorescence staining to detect the expression and localization of PTRF, and we found that UBE2O overexpressed cells have less cell edge-localized PTRF (Fig. 5A). In line with the important roles of PTRF in caveolae formation [21–25], transmission electron microscopy (TEM) scanning was performed to show the caveolae abundance. TEM images and the statistical data showed that PTRF overexpressed cells have more caveolae per micrometer of cell membrane compared to untransfected cells. Importantly, UBE2O restricts caveolae formation in both empty cells and PTRF expressing cells (Fig. 5B, C). Based on caveolin-1 (CAV1) is also responsible to drive caveolae formation and CAV1 interacts with PTRF in the condition of lipid raft integrity [47], we wonder whether UBE2O disrupts the interaction between CAV1 and PTRF. The results showed that UBE2O did not disrupt the interaction between CAV1 and PTRF, but decreased the expression level of CAV1 with the decreased expression of PTRF (Additional file 2: Figure S3A). Similarly, we also found that the expression level of CAV1 was reduced in PTRF knockdown HeLa cells (Additional file 2: Figure S3B and C). Interestingly, we did not find a strong interaction between CAV1 and UBE2O in HeLa cells and U-2 OS cells, whereas there was a strong interaction between CAV1 and UBE2O in U251 cells, a PTRF high cell line (Additional file 2: Figure S1B, C and S3D). Since the cell membrane invagination is the first step of exosome secretion [2, 3], these data illustrated that UBE2O controlled the secretion of exosome-related PTRF through reduced PTRF in caveolae and limited caveolae formation.

### UBE2O knockout increases the secretion of exosome-related PTRF and upregulates exosome markers

**Fig. 6:**
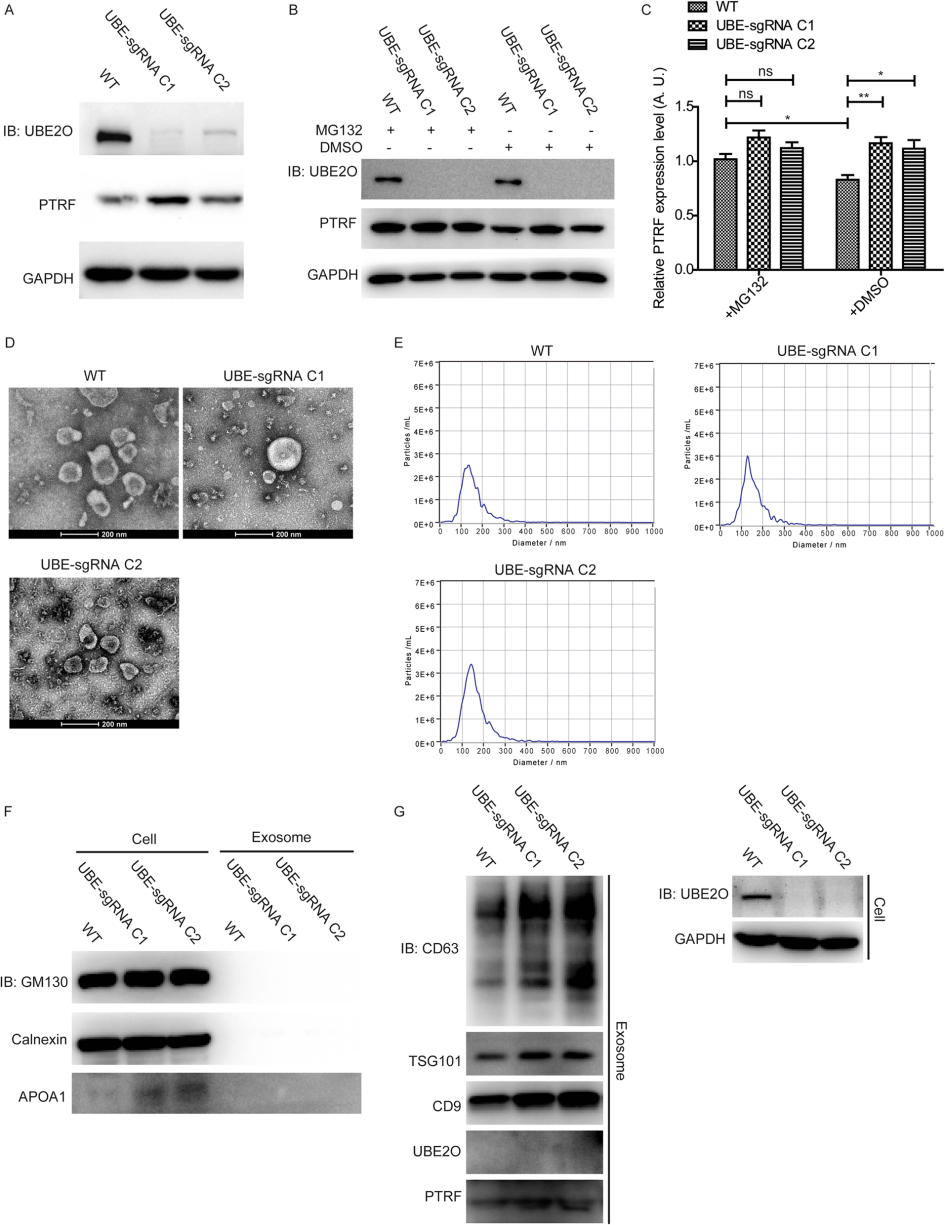
UBE2O knockout upregulates PTRF expression in both cells and exosomes. **A** Western blots analysis compared the expression levels of PTRF in wild type and two clones of UBE2O KO HeLa cells. **B** Wild type and UBE2O KO HeLa cells were treated with 10 μM MG132 and DMSO for 4 h before the cell collection. Then, the expression level of PTRF in cells was analyzed by western blot. **C** Densitometry analyses of PTRF expression relative to GAPDH expression for the result of western bolt in Fig. 6B were shown by the graph. Data are the means $\pm$ SEM, n = 4. ns means non-significant, *P $<$ 0.05; **P $<$ 0.01. **D** After wild type and two clones of UBE2O KO HeLa cells were cultured in serum-free culture medium for 40 h, the supernatants were used for exosomes isolation. Then, exosomes harvested from supernatants were identified by transmission electron microscopy. **E** Exosome samples from wild type and two clones of UBE2O KO HeLa cells were diluted in appropriate concentration to analyzed by NTA. **F**, **G** Exosomes from wild type and two clones of UBE2O KO HeLa cells were analyzed by western blot for the indicated antibodies

We constructed two clones of UBE2O knockout cell lines, and found that the expression of PTRF was induced in UBE2O knockout cell lines (Fig. 6A). To study the degradation pathway for the ubiquitinylated PTRF, the proteasome inhibitor, MG132, was used to treat the wild type and UBE2O knockout cells. As shown in Fig. 6B and C, parts of PTRF expression in the normal cells were rescued by MG132, illustrating that the proteasomal degradation pathway was not the unique pathway to degrade PTRF via UBE2O. Moreover, exosomes which were isolated from the supernatants of wild type and UBE2O knockout HeLa cell lines were identified by TEM, NTA, negative and positive exosome markers (Fig. 6D, E, F, G). In addition, western blot analysis for exosomes showed that PTRF content and all exosome markers were increased in exosomes in the UBE2O knockout cell line groups, compared with that in the control group (Fig. 6G).

### SDPR promotes PTRF secretion via exosomes and does not block the effect of UBE2O on the secretion of exosome-related PTRF

**Fig. 7:**
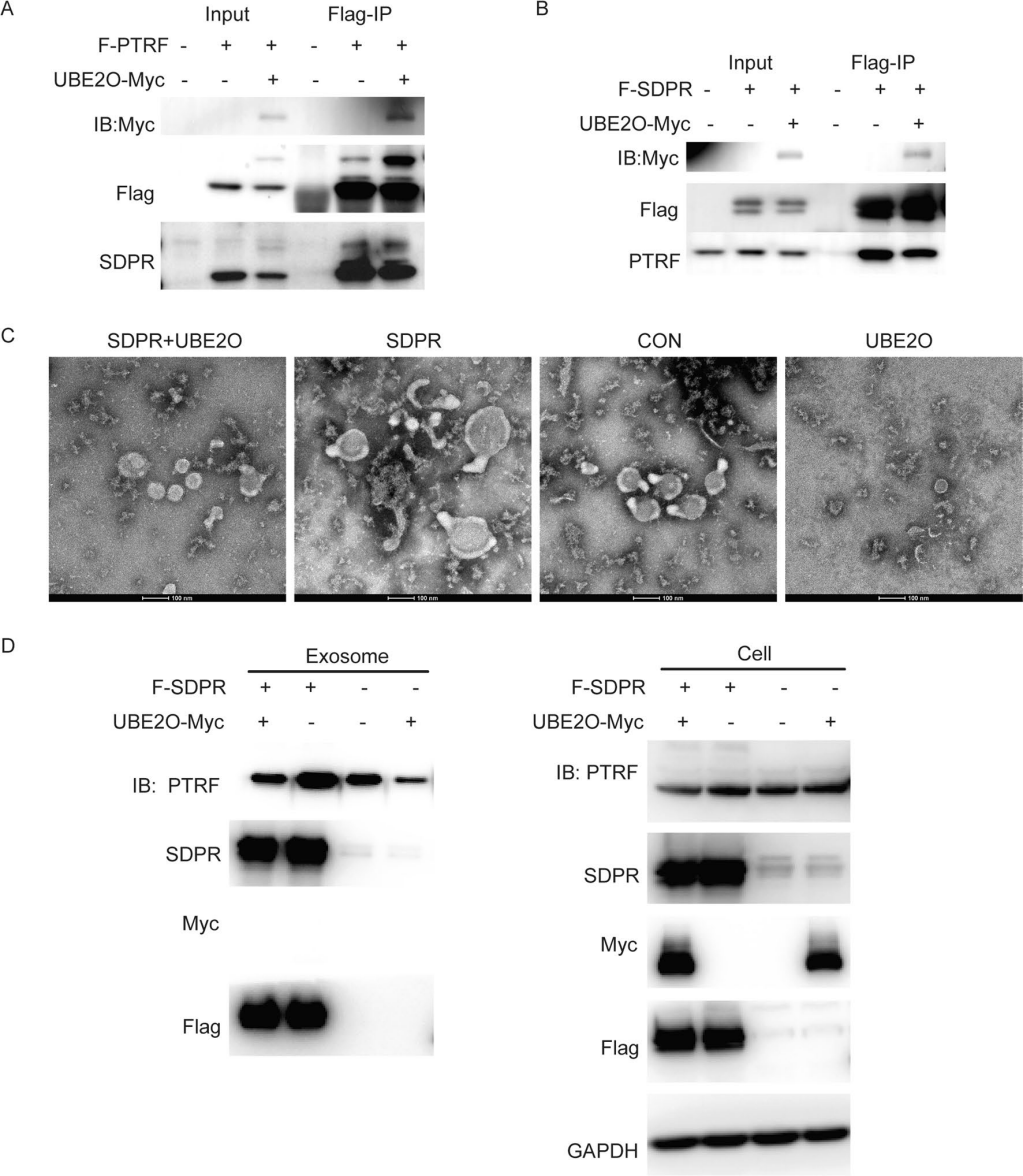
SDPR stabilizes PTRF secretion via exosomes and does not impede the effect of UBE2O. **A** Flag-PTRF and UBE2O-Myc were overexpressed in HeLa cells. Then, Flag-PTRF in the cell lysates was immunoprecipitated with Flag M2 beads. Flag-PTRF associated endogenous SDPR in HeLa cells was immunoblotted by western blot. **B** Flag-SDPR and UBE2O-Myc were co-transfected into HeLa cells. Flag-SDPR in the cell lysates was immunoprecipitated with Flag M2 beads. The immunoprecipitates and the total cell lysates were immunoblotted for the indicated antibodies. **C**, **D** UBE2O-Myc was transiently transfected into Flag-PTRF stable HeLa cell line or Flag-SDPR stable HeLa cell line. Serum-free culture medium which had culture HeLa cell lines for 40 h was collected to isolate exosomes. Then, exosomes were identified by transmission electron microscopy (**C**). Cell lysates and exosomes were analyzed by western blot as indicated (**D**)

A previous study showed that SDPR interacts with PTRF [26]. In addition, our results have demonstrated that UBE2O interacts with both PTRF and SDPR. To identify the role of UBE2O in the interaction between SDPR and PTRF, we further did semi-endogenous immunoprecipitation assays with Flag-PTRF and Flag-SDPR in the condition of UBE2O overexpression respectively, which found that UBE2O reduced the interaction between PTRF and SDPR instead of disrupting the combination of PTRF and SDPR because either Flag-SDPR-associated endogenous PTRF or Flag-PTRF-associated endogenous SDPR was only slightly decreased (Fig. 7A, B). It was reported that SDPR stabilized PTRF and recruited PTRF to caveolae in HeLa cells [26]. To address whether SDPR was required for PTRF stable secretion via exosomes, the exosomes which were obtained from the SDPR overexpression cell line were identified by TEM (Fig. 7C) and analyzed by western blot. The results of SDPR overexpression showed that the stable secretion of PTRF via exosomes also required SDPR, which was also consistent with the results of SDPR knockdown (Fig. 7D, Additional file 2: Figure S4A, B). Interestingly, with the overexpression of UBE2O in cells, the expression of PTRF also decreased in exosomes of SDPR overexpression cell line (Fig. 7D). Taken together, SDPR did not impede the effect of UBE2O on the secretion of exosome-related PTRF, although SDPR induced PTRF secretion via exosomes.

## Discussion

UBE2O is a large E2 enzyme, which has both E2 and E3 ubiquitin-protein ligase activities [28, 29]. UBE2O ubiquitinates with and degrades a variety of proteins, such as the human RecQ DNA helicase (RECQL4), MAX interactor 1 (Mxi1) and c-Maf [33, 40, 48]. It is reported that UBE2O also interacts with more than 100 E3 ubiquitin-protein ligases. Thus, UBE2O can mediate various functions in cells depending on its function of ubiquitination. It is necessary to find out the proteins which were ubiquitinated by UBE2O. Nguyen, et al. showed that UBE2O ubiquitinated PTRF during terminal erythroid differentiation [45]. Nevertheless, how UBE2O interacts with PTRF and how UBE2O ubiquitinates PTRF have not been mentioned. Our results showed that PTRF directly interacted with UBE2O via the domains including C-terminal NLS, PEST sequences and LZ. Moreover, Liu et al. have mentioned that mouse PTRF is mono-ubiquitinated [49]. Here, we identified that UBE2O ubiquitinated human PTRF via both monoubiquitination and polyubiquitination. Future research needs to explore the type of UBE2O mediated polyubiquitination of PTRF.

PTRF has a critical role in caveolae formation and has been mentioned to mediate exosome release [18, 21–25]. Notably, previous studies reported that Corola controlled the biogenesis of extracellular vesicles through neddylated by UBE2F and TRIM4 [50]. In addition, decreasing the degradation of Rab27a by the ubiquitin–proteasome pathway limits exosome secretion [32]. These studies provide evidences that neddylation and ubiquitination affect extracellular vesicles release, and E2 and E3 enzymes are related to extracellular vesicle secretion closely. Since we demonstrated that UBE2O ubiquitinated PTRF, we wonder whether UBE2O mediates exosome secretion. Here, we found that UBE2O inhibited caveolae formation, resulting in the reduction of exosome release. Our data first showed that UBE2O had the function to mediate exosome secretion.

It is demonstrated that most tumor-derived exosomes were beneficial for tumor progression [51, 52]. Exosomes derived from HeLa cells facilitated tumor metastasis through breaking down vascular integrity had been reported [14]. Our results provided evidence that UBE2O reduced exosomes release in HeLa cells. Thus, activating UBE2O might be a potential therapy for HeLa cell-associated cervical cancer. However, UBE2O has two sides in different types of cancers. UBE2O inhibits myeloma tumor growth, while it promotes tumorigenesis in breast cancer and prostate cancer by degradation of AMPKα2 and facilitated lung cancer progression by degradation of Mix1 [36, 48]. Nevertheless, if there was no AMPKα2 or Mix1 expression in cancer cells, ablation of UBE2O expression did not display dramatically anti-tumor effects. Here, we did not detect the expression quantity of AMPKα2 and Mix1 in HeLa cells.

Previous studies demonstrated that PTRF expression in both serum exosomes and tumor samples was related to glioma grade [18]. Moreover, clear cell renal cell carcinoma (ccRCC) is the typical subtype of renal cell carcinoma (RCC) [53]. The expression of PTRF in ccRCC cancer cells and ccRCC cancer cell-derived exosomes was increased, indicating that PTRF in urine exosomes could be a promising biomarker of ccRCC [19]. Because UBE2O restricted reduced caveolae formation and exosome release through ubiquitinated PTRF, we wonder whether UBE2O regulates the secretion of exosome-related PTRF. Interestingly, our results showed that UBE2O dramatically reduced the secretion of exosome-related PTRF, suggesting that an increase of UBE2O expression might be an approach to treat glioma and ccRCC. Here, our data also first provided an approach for regulating the secretion of exosome-related PTRF. In consideration of UBE2O downregulated exosome release and inhibited the secretion of exosome-related PTRF, it is also indirectly demonstrated that UBE2O limited PTRF-loaded exosome secretion. However, the characteristic of PTRF in exosomes is poorly understood.

SDPR was identified to directly bind to PTRF [26], and we have demonstrated that PTRF interacted with UBE2O directly in this study. In addition, our previous UBE2O interactome study also identified SDPR [40]. To explore the relation between SDPR and UBE2O, we performed immunoprecipitation assays and showed that SDPR combined with the UBE2O CR2 domain, which was the same as PTRF. Nevertheless, how SDPR, PTRF and UBE2O cooperate to regulate exosome secretion is still poorly understood. Caveolae were distended and elongated when SDPR was overexpressed in cells. SDPR also expanded the area of caveolae. Interestingly, we were surprised to find that UBE2O could rescue these phenomena (Additional file 2: Figure S5A, B and C). Considering that SDPR was secreted with exosomes, it seems there is a role for SDPR in exosome secretion. Since SDPR is related to the morphology of caveolae, whether SDPR changes the proportion of the different sizes of extracellular vesicles and whether UBE2O alters the size of extracellular vesicles via SDPR also can be the future research.

Hansen et al. demonstrated that SDPR stabilized PTRF expression in cells and recruited PTRF to caveolae [26]. To study how SDPR modulates the secretion of exosome-related PTRF, first of all, we performed immunoprecipitation assay in the existence of UBE2O, and identified that UBE2O did not completely disrupt the combination of SDPR and PTRF. Then, we overexpressed SDPR in cells and collected exosomes. Interestingly, we found that SDPR induced PTRF secretion via exosomes, which revealed that the inhibition of SDPR may be another approach to regulating the secretion of exosome-related PTRF in cancer cells excepted UBE2O. Moreover, UBE2O overexpressed in the SDPR stable cell line still dramatically inhibited the secretion of exosome-related PTRF, which illustrated that SDPR also did not absolutely disrupt the interaction of PTRF and UBE2O. Importantly, there was no UBE2O included in exosomes even though UBE2O modulated exosome secretion, thus UBE2O may indirectly participate in the processes of exosome biogenesis.

## Conclusions

UBE2O degraded PTRF by ubiquitination, leading to reduce the formation of caveolae. Exosome biogenesis which originated from caveolae was inhibited, leading to the reduction of exosome release. Importantly, these processes ultimately resulted in UBE2O controlling the secretion of exosome-related PTRF. Moreover, SDPR increased PTRF secretion via exosomes. Notably, UBE2O still downregulated the secretion of exosome-related PTRF in the condition of SDPR overexpression. Our results provide a novel approach for regulating the secretion of exosome-related PTRF and provide a novel mechanism of how UBE2O mediated ubiquitination regulates exosome secretion.

## Supplementary Information

**Additional file 1**. The sequences of primers.

**Additional file 2**. Supplemental figures.

## Data Availability

All datasets used and/or analysed during the current study are available from the corresponding author on reasonable request.

## Abbreviations

MVBs: Multivesicular bodies
PTRF: Polymerase I and transcript release factor
UBE2O: Ubiquitin-conjugating enzyme E2O
SDPR: Serum deprivation protein response
ILVs: Intraluminal vesicles
AMPKα2: AMP-activated protein kinase-α2
MLL: Mixed-lineage leukemia
ARNTL (BMAL1): Aryl hydrocarbon receptor nuclear translocator-like protein 1
NLS: Nuclear localization sequence
LZ: Leucine-zipper motif
PEST: PEST sequence
CR2: The conserved region 2
TEM: Transmission electron microscopy
NTA: Nanoparticle tracking analysis
RECQL4: The human RecQ DNA helicase
Mxi1: MAX interactor 1
ccRCC: Clear cell renal cell carcinoma
RCC: Renal cell carcinoma
FBS: Fetal bovine serum
PEI: Poly-ethylenimine
α-TG: α-Thioglycerol
BCS: Bathocuproinedisulfonic acid disodium salt
NEM: N-Ethylmaleimide
IPTG: Isopropyl thiogalactoside
PBS: Phosphate-buffered saline
PFA: Paraformaldehyde
DAPI: 4’, 6-Diamidine-2-phenylindole
