# Supplementary material for: UBE2O ubiquitinates PTRF/CAVIN1 and inhibits the secretion of exosome-related PTRF/CAVIN1
Source: Cell Commun Signal. 2022 Nov 28;20:191. doi: 10.1186/s12964-022-00996-z (PMC9703712; doi:10.1186/s12964-022-00996-z)
Supplement: Supplementary file 2 — Additional file 1. The sequences of primers. [file 12964_2022_996_MOESM2_ESM.docx]

**Supplementary information**

**Table S1. The names of primer and their sequences.**

| The name of primer | sequence |
| --- | --- |
| UBE2O deletion constructs D1 forward primer | atggcggatcccgcagccccca |
| UBE2O deletion constructs D1 reverse primer | agtcatgttcttgaggctctccagg |
| UBE2O deletion constructs D2 forward primer | ctggagagcctcaagaacatgactg |
| UBE2O deletion constructs D2 reverse primer | cttgtcctctgtgcactccggca |
| UBE2O deletion constructs D3 forward primer | atggcggatcccgcagccccca |
| UBE2O deletion constructs D3 reverse primer | ctgcaggcctcggtcactgtcg |
| UBE2O deletion constructs D4 forward primer | gcggagtctgccagccctgag |
| UBE2O deletion constructs D4 reverse primer | cttgtcctctgtgcactccggca |
| UBE2O deletion constructs D5 forward primer | gggccgtcctcggactccgg |
| UBE2O deletion constructs D5 reverse primer | cttgtcctctgtgcactccggca |
| PTRF deletion constructs D1 forward primer | atggaggaccccacgctctatat |
| PTRF deletion constructs D1 reverse primer | gcgctccaggctgccg |
| PTRF deletion constructs D2 forward primer | atgcaggcggggcagatcaaga |
| PTRF deletion constructs D2 reverse primer | acgctctgcgcgggactcctc |
| PTRF deletion constructs D3 forward primer | atgatcaagcgcagcggcct |
| PTRF deletion constructs D3 reverse primer | gtcgctgtcgctcttgtccac |
| SDPR deletion constructs D1 forward primer | atgggagaggacgctgcacag |
| SDPR deletion constructs D1 reverse primer | caaatcatcatctgaggagaggtcc |
| SDPR deletion constructs D2 forward primer | atgccccacgatgaggaggc |
| SDPR deletion constructs D2 reverse primer | ggaggtctggtgcacctggag |
| The truncated version of UBE2O CR2 forward primer | gcggagtctgccagccctgag |
| The truncated version of UBE2O CR2 reverse primer | agtcatgttcttgaggctctccagg |
| UBE2O sgRNA forward primer | caccgcatctatcccgtcaacagca |
| UBE2O sgRNA reverse primer | aaactgctgttgacgggatagatgc |
| PTRF-shRNA-1- forward primer | ctagacaagagcgacagcgactgagctcgagctcagtcgctgtcgctcttgttttttc |
| PTRF-shRNA-1- reverse primer | tcgagaaaaaacaagagcgacagcgactgagctcgagctcagtcgctgtcgctcttgt |
| PTRF-shRNA-2- forward primer | ctaggagcatcagcaaatcgctgaactcgagttcagcgatttgctgatgctctttttc |
| PTRF-shRNA-2- reverse primer | tcgagaaaaagagcatcagcaaatcgctgaactcgagttcagcgatttgctgatgctc |
| PTRF-shRNA-3- forward primer | ctagcctactagaaggacgtgaaagctcgagctttcacgtccttctagtaggtttttc |
| PTRF-shRNA-3- reverse primer | tcgagaaaaacctactagaaggacgtgaaagctcgagctttcacgtccttctagtagg |
| PTRF-shRNA-4- forward primer | ctagcgaacttcctctttcgcattcctcgaggaatgcgaaagaggaagttcgtttttc |
| PTRF-shRNA-4- reverse primer | tcgagaaaaacgaacttcctctttcgcattcctcgaggaatgcgaaagaggaagttcg |
| SDPR-shRNA-1- forward primer | ctaggaatagtgtaccggtaatatactcgagtatattaccggtacactattctttttc |
| SDPR-shRNA-1- reverse primer | tcgagaaaaagaatagtgtaccggtaatatactcgagtatattaccggtacactattc |
| SDPR-shRNA-2- forward primer | ctagcagcaacatcgacttgactatctcgagatagtcaagtcgatgttgctgtttttc |
| SDPR-shRNA-2- reverse primer | tcgagaaaaacagcaacatcgacttgactatctcgagatagtcaagtcgatgttgctg |
| SDPR-shRNA-3- forward primer | ctagcaagctggtgaacatgctagactcgagtctagcatgttcaccagcttgtttttc |
| SDPR-shRNA-3- reverse primer | tcgagaaaaacaagctggtgaacatgctagactcgagtctagcatgttcaccagcttg |
| Human UBE2O for qPCR- forward primer | tgcatcatctatcccgtcaaca |
| Human UBE2O for qPCR- reverse primer | ccagcagtcataggcaatgtag |
| Human PTRF for qPCR- forward primer | aggtcagcgtcaacgtgaag |
| Human PTRF for qPCR- reverse primer | ccgactctttcagcgatttgc |
| Human TSG101 for qPCR- forward primer | gagagccagctcaagaaaatgg |
| Human TSG101 for qPCR- reverse primer | tgaggttcattagttccctgga |
| Human CD63 for qPCR- forward primer | atgcaggcagattttaagtgct |
| Human CD63 for qPCR- reverse primer | gttcttcgacatggaagggattt |
| Human CD9 for qPCR- forward primer | cctgctgttcggatttaacttca |
| Human CD9 for qPCR- reverse primer | tggtctgagagtcgaatcgga |
| Human GAPDH for qPCR- forward primer | gagtcaacggatttggtcgt |
| Human GAPDH for qPCR- reverse primer | gacaagcttcccgttctcag |

The names of primer and their sequences, which have been used in this study were listed above.
