# Supplementary material for: UBE2O ubiquitinates PTRF/CAVIN1 and inhibits the secretion of exosome-related PTRF/CAVIN1
Source: Cell Commun Signal. 2022 Nov 28;20:191. doi: 10.1186/s12964-022-00996-z (PMC9703712; doi:10.1186/s12964-022-00996-z)
Supplement: Supplementary file 3 — Additional file 2. Supplemental figures. [file 12964_2022_996_MOESM3_ESM.docx]

**Supplemental information**


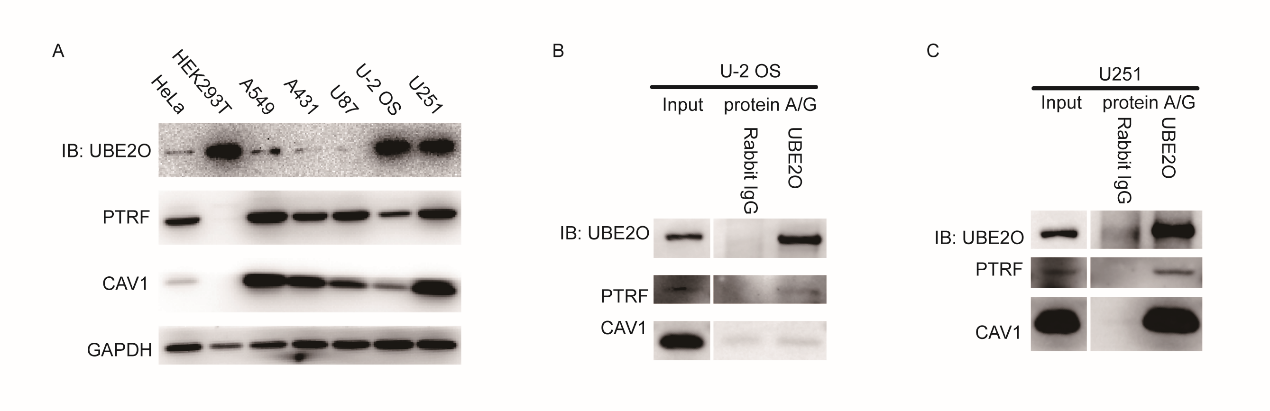
**Figure S1 UBE2O interacts with PTRF endogenously in U-2 OS cells and U251 cells.** (A) The expression level of PTRF in HeLa cells, HEK293T cells, A549 cells, A431 cells, U87 cells, U-2 OS cells and U251 cells was analyzed by western blots. (B, C) Endogenous UBE2O in U-2 OS cells (B) or U251 cells (C) was immunoprecipitated with UBE2O antibody by protein A/G. UBE2O-associated endogenous PTRF and UBE2O-associated CAV1 were detected by IB. To ensure the results were visualized, HRP, Rabbit IgG light chain secondary antibody was used for the western blot of PTRF to eliminate heavy chain interference.


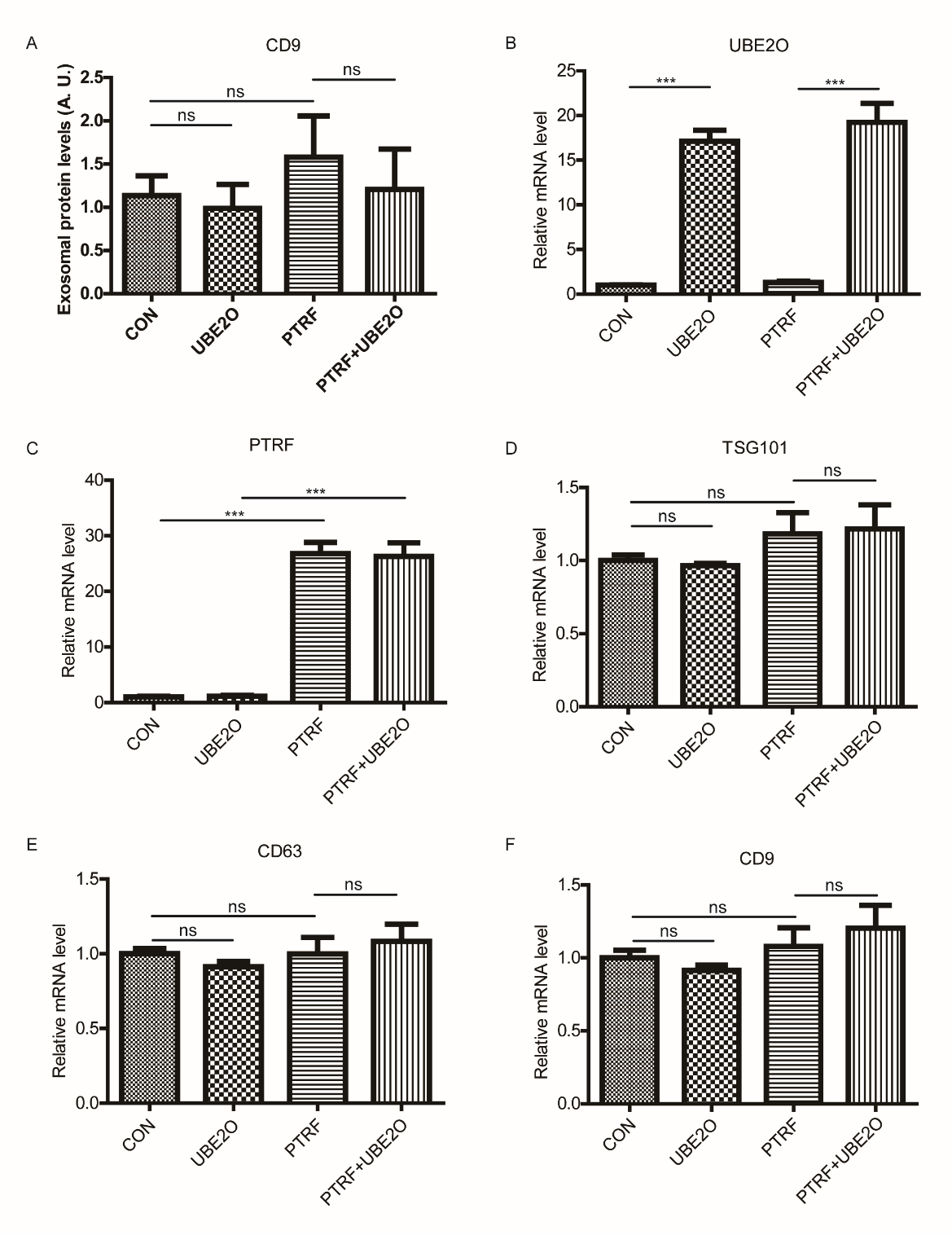
**Figure S2 UBE2O did not change exosome-related CD9 secretion and RNA level of exosome markers in cells.** (A) Densitometry analysis of exosomal CD9 expression relative to cell GAPDH expression was shown by histogram. (B, C, D, E, F) The relative mRNA levels of UBE2O (B), PTRF (C), TSG101 (D), CD63 (E) and CD9 (F) in UBE2O or PTRF overexpression HeLa cells were evaluated by quantitative RT-PCR. n=4; ns means non-significant; ****P* $<$0.001.

**
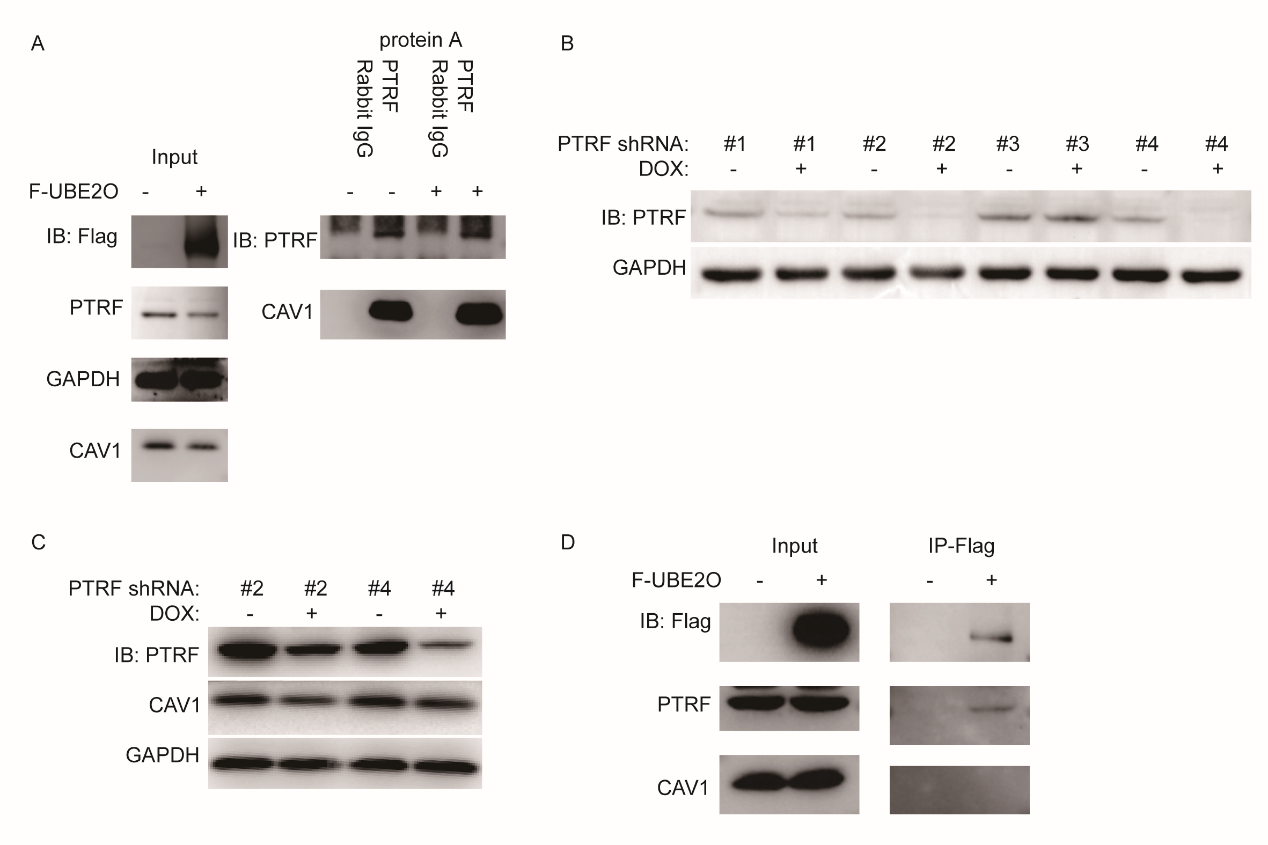
Figure S3 The expression of CAV1 was downregulated with the decreased expression of PTRF.** (A) HeLa cells were transfected with Flag-UBE2O for 36h. After cell lysates collection, PTRF-associated endogenous CAV1 was detected by IP and IB. (B) Inducible PTRF knockdown HeLa cell lines were with or without 200 ng/ml doxycycline treatment for 24h before cell lysates collection. The expression level of PTRF in PTRF knockdown cell lines was shown by western blot. (C) PTRF knockdown cell lines which expressed PTRF shRNA2 and PTRF shRNA4 separately were induced by 200 ng/ml doxycycline for 24h. The expression levels of PTRF and CAV1 in PTRF knockdown cell lines were detected by western blot. (D) Flag-UBE2O expression plasmid was transfected into HeLa cells. After 36h transfection, cell lysates were harvested. Flag-UBE2O in cell lysates was immunoprecipitated with Flag M2 beads. IB was used to analyze the immunoprecipitates of Flag-UBE2O and the total cell lysates.

**Figure S4 The secretion of exosome-related PTRF was reduced with the knockdown of SDPR.** (A) 200 ng/ml doxycycline was used to treat inducible SDPR knockdown HeLa cell lines for 24h. The cell lysates were harvested and the expression level of SDPR in cells was analyzed by western blot. (B) Inducible SDPR knockdown HeLa cell lines were cultured in serum-free culture medium with or without 200 ng/ml doxycycline for 48h. Cells were lysed by RIPA lysis buffer and the serum-free culture medium was harvested to isolate exosomes. Cell lysates and exosomes were analyzed by the indicated antibodies via western blot.


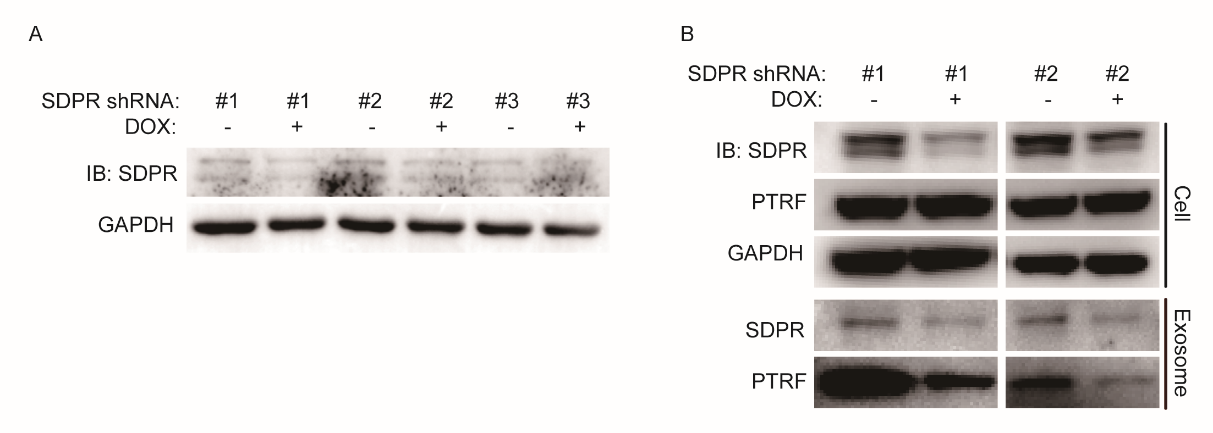


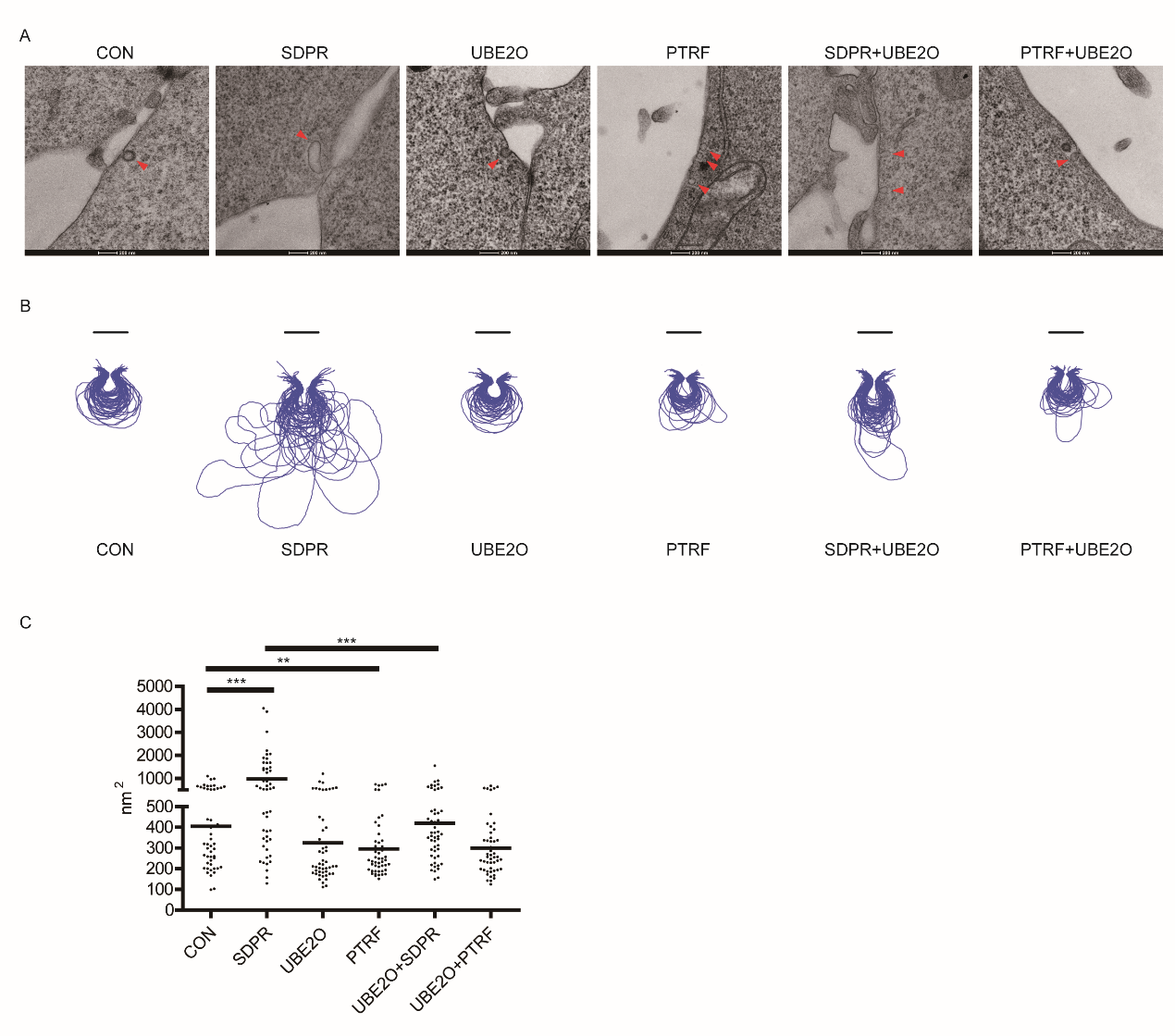
**Figure S5 Morphology of caveolae is rescued by UBE2O in the condition of SDPR overexpression.** (A) UBE2O-Myc was transfected into Flag-SDPR stable cell line or Flag-PTRF stable cell line. Pictures of caveolae structures were shown by TEM scanning. Red arrowhead: caveolae, Scale bar: 200nm. (B) 50 caveolae from each group of overexpression cell line were manually superimposed. Bars are 100nm. (C) Statistical analysis for the areas of 50 caveolae from each group of overexpression cell line was performed. *P* values were calculated with Student’s t-test. ***P* $<$0.01, ****P* $<$0.001.
